# Cordymin alleviates osteoporosis induced by hindlimb unloading via regulating the gut - microelements -bone axis --for non-clinical studies

**DOI:** 10.1186/s12891-023-07057-7

**Published:** 2023-12-01

**Authors:** Wei Qi, Tiancheng Ma, Yufei Ji, Hong Jia, Qiang Sun, Dawei Zhang

**Affiliations:** 1 Department of Orthopaedics, Xijing Hospital, The Air Force Medical University, Xi’an, 710032 China; 2 Xi’an, China

**Keywords:** Cordymin, Gut microbiome, Microelement, Hindlimb unloading

## Abstract

**Introduction:**

The purpose of this study was to evaluate the protective effects of cordymin on osteoporosis induced by hindlimb unloading(HLU) in rats and whether cordymin can prevent bone loss from HLU.

**Materials and methods:**

We employed the hindlimb suspension rats model to mimic physiological changes concomitant with space travel.The mechanical strength in the femoral neck,cancellous bone volume, gut microbiota structure,serum calcium and phosphorus contents, bone mineral content and bone mineral content can be changed after hindlimb unloading. Oral cordymin was administered for 4 weeks,cordymin treatment significantly increased the mechanical strength through elevated bone volume/tissue volume (BV/TV), trabecular number (Tb. N), trabecular thickness (Tb. Th) and decreased trabecular separation (Tb. Sp).

**Results:**

Importantly, 16 S rRNA sequencing showed cordymin treatment regulated the various genera that were imbalanced in hindlimb unloading rats. At the same time,The plasma total calcium and inorganic phosphate concentrations in hindlimb unloading rats decreased and bone mineral content in the lumbar vertebrae and femur increased after treatment with cordymin.

**Conclusion:**

These data indicate that the cordymin might exert bone protective effects indirectly via modulating the complex relationship between gut microbiota, microelements and bone loss.

## Introduction

Microgravity, or weightlessness, is one of the main problems that astronauts have to cope with during space missions, which can cause changes in the balance between osteoblasts and osteoclasts. It has been reported that astronauts experienced 1.5–2% loss of bone mass each month in the microgravity environment [1]. Take microgravity-induced bone loss in the mechanical unloading environment of space, bone resorption increases significantly,while bone formation remains no change or possibly decreased [2]. This imbalance leads to 1–1.5% bone mass loss per month. Microgravity-induced osteoporosis threatens the safety and health of astronauts during space flight. Astronauts are at risk of progressive bone loss in space and the space-related bone and joint changes may persist for years after their return to Earth [3]. Current research, although limited, revealed the linkages between gut microbiota and bone metabolism is maintained through a combination of mineral absorption. Therefore, further exploration of this relationship is a promising area for bone health and osteoporosis research.

Currently, the role of such trace elements as magnesium, copper, zinc, manganese, cadmium, and lead in the development of osteoporosis and the effects of treating mineral profiles on osteoporosis have yet to be fully elucidated [4]. Metal elements in the body can affect bone mineral formation and bone matrix synthesis [5]. The “Microelements-Bone axis” affect the synthesis and cross link of bone organic collagen fiber, which plays a vital role in the growth and development of bone [6].

At the same time, there are evidence shows the relationship between intestinal flora and bone metabolism [7]. Several studies have found that depletion of the gut microbiota significantly improved decreased bone mass. This evidence suggests that the gut microbiome may furnish effective novel biomarkers in the diagnosis/prognosis of bone disease and other phenotypic traits [8]. Further studies have confirmed gut microbiome or their metabolites as a linker in the “Gut-Bone axis” [9].

Minerals such as calcium, iron, zinc, magnesium, and phosphorous can also shape the human gut microbiome [10]. On the other hand, gut microbiome affects the mineral metabolism, which are exclusive for the colonic microbes and that help releasing minerals from foods [11]. This relationship between metal trace elements and gut microbiome resulted in the formation of “Gut-Microelements axis”.

The“Gut-Bone axis”, “Microelements-Bone axis” and “Gut-Microelements axis”play a vital role in the growth and development of bone [6, 9, 10]. Nevertheless, few studies had been carried out on the crosstalk between microelement and gut microbiome of osteoporosis. The “Gut-Microelements-Bone axis” will be an important target for treatment of disuse osteoporosis. Our previous research results confirmed that administration of cordymin, a peptide purified from the *Cordyceps sinensis*, has significant effects in rat model of diabetic osteopenia. Cordymin could restore the circulating blood glucose, glycosylated hemoglobin (HbA1c), serum alkaline phosphatase (ALP), tartrate resistant acid phosphatase (TRAP), and insulin levels in a dose-dependent manner, as well as increased bone mineral content (BMC) and bone mineral density (BMD) in diabetic rats [12]. However, there is little amount of information available about cordymin for treatment of osteoporosis induced by hindlimb unloading. The HLU model has been widely used to simulate the microgravity environment. Several different techniques have been used to induce hindlimb unloading in rodents over the past four decades [13]. The present study aimed to investigate whether cordymin can prevent bone loss from HLU and the underlying mechanism of its action via regulating “Gut-Microelements-Bone axis”.

## Materials and methods

### Animals

Fifty healthy male Wistar rats(2 months old and weighing 225 ± 25 g) ,provided by the Laboratoey Animal Center of Air Force Medical University, were used in the study.

The rats were randomly divided into five groups by SPSS software [14], including the control group,the hindlimb suspension group, the hindlimb suspension with cordymin-25 group,the hindlimb suspension with cordymin-50 group and the hindlimb suspension with cordymin-100 group. The control group was treated with vehicle (0.9% saline) but without hindlimb suspension, while both the hindlimb suspension group and the cordymin groups were hindlimb unloaded by suspending their tails for 28 days. The rats in the cordymin treatment group were treated orally with cordymin (25, 50, and 100 mg/kg/day) for 5 weeks, and the other 2 groups of rats were treated orally with saline (6 mL/kg/day). The studies involving animals were reviewed and approved by ethics committee of the First Affiliated Hospital of the Military Medical university of Air-force of PLA as well as ARRIVE 2.0. Care was taken to minimize discomfort, distress, and pain to the animals.

### Preparation of cordymin

Cordyceps sinensis was collected from Qing Hai, China. Cordymin was prepared according to our previous way [12]. Briefly, dried fruiting bodies of Cordyceps sinensis (100 g) were homogenized in liquid nitrogen with a pestle, extracted in distilled water and centrifuged. To the resulting supernatant, ammonium acetate buffer (pH 4.5) was added until a final concentration of 20 mM was attained. The sample was loaded on an SPSepharose column. The adsorbed fraction was eluted with 1 M NaCl in 20 mM ammonium acetate buffer (pH 4.5), then dialyzed against distilled water, and lyophilized. Then it was dissolved in 20 mM NH4OAc buffer (pH 4.5) and applied on a Mono S column and eluted with the same buffer. The fraction containing cordymin was concentrated and then purified on a Superdex 75 column in the same buffer. The single peak eluted constituted purified peptide designated as cordymin. The extracts were stored at -30℃ before use.

### Suspension procedure

The tail suspension method utilized in the present study was the same as the method used by Morey-Holton et al [15]. Briefly,the tail was inserted into a harness affixed to a guide wire running the length of the top of the cage. The height was adjusted to ensure each animal had its hindlimbs elevated and with a head-down tilt of 35–40. The forelimbs of rats were free and reached the ground. Therefore, they could move around the cage and had complete access to food and water. Body weight was recorded daily. Control rats were housed individually in similar cages located in the same room but had no attachments to the tail. The rats were maintained at a constant temperature of 25℃, and kept on a 12-hour light/dark cycle during the experimental procedure. On the last day of treatment the fecal samples of 5 rats were taken with sterile EP tube. All fecal samples were stored at − 80℃. Blood samples were collected and centrifuged at 3000 rpm for 10 min to separate serum for further analysis of trace elements. The left femur and L-4 vertebra bone were processed for mineral content measurement and histopathological examination. The distal femurs were processed forµCT.

### Measurement of mechanical properties

Applying a vertical load to the femoral head using a Shimadzu EZ-1 pressure system (Shimadzu, Osaka, Japan), the mechanical strength including ultimate compressive load (newton) was measured. The fracture load was recorded at the peak force as Newton (N) at the point that the femoral neck fractured.

### µCT analysis of the distal femurs

The steps involved in the evaluation of bone morphometry and tissue mineral density by mCT, namely, image acquisition, image processing, image analysis, and reporting of results according to current guideline [16]. Distal Femurs were scanned at 48-µm intervals using an experimental animal CT system (LaTheta LCT-200; Hitachi Aloka Medical, Tokyo, Japan). Bone volume (BV) was calculated using tetrahedrons corresponding to the enclosed volume of the triangulated surface. Total volume (TV) was the volume of the sample that was examined. A normalized index, BV/TV, was utilized to compare samples of varying size. The methods used for calculating trabecular thickness (Tb. Th), trabecular separation (Tb.Sp), trabecular number (Tb.N) and structural model index (SMI) have been described by Lane NE. Cortical thickness (Ct.Th) was expressed as the average cortical thickness of three Sect. [17].

### Histopathological examination of bone tissues

Four micrometer-thick sections of femurs were cut by the aid of a microtome and stained with hematoxylin and eosin (H&E). The femurs were decalcifed using 10% ethylenediaminetetraacetic acid (EDTA; pH = 7.4) for 1 month. The EDTA solution was replaced every 2 days. The decalcified femurs were washed, dehydrated, and embedded in paraffin. Paraffin-embedded sections were prepared, dewaxed, and rehydrated. Five micrometer sections were cut and stained with standard H&E. The tissue specimens were examined for arrangement of bone marrow trabecula and intertrabecular spaces. Three representative sections (top, middle, and bottom levels of the specimen) were analyzed per animal. The mean value of the fields in the three sections measured for each animal was used for statistical analysis.

### DNA extraction of feces

Genomic DNA was extracted and purified from feces and periodontal ligatures with the HiPure Stool DNA Kits (Magen, Guangzhou, China) according to the manufacturer’s protocols. The resulting DNA was quantified with Quant-iTTM PicoGreen reagent (Magen, Guangzhou, China).

### DNA sequencing and analyses

The V3-V4 regions of the 16 S rRNA were amplified with the MiSeq 300PE (Illumina MiSeq System). The primers is (341 F: CCTACGGGNGGCWGCAG and 806R: GGACTACHVGG-GTATCTAAT). Subsequently, purified amplicons were pooled in equimolar amounts, and paired-end sequenced on an Illumina platform according to standard protocols described by Gene Denovo Biotechnology Co., Ltd (Guangzhou, China). The PCR cycling conditions were as follows: initial denaturation at 95 °C for 3 min, followed by 27 cycles of denaturing at 95 °C for 30 s, annealing at 55 °C for 30 s, and extension at 72 °C for 45 s, with a final extension at 72 °C for 10 min. Raw reads of 16 S rRNA gene sequences were de-multiplexed and quality-filtered using FLSAH. Chimeric sequences were identified and removed using UCHIME. By cutting and filtering reads and clustering operational taxonomic units (OTUs), species annotation and abundance analysis can reveal the species composition of samples. The α diversity analysis and β diversity analysis were used to reveal differences between samples [18].

### Bone mineral content measurement

In order to determine the content of mineral substances in bones, the left femur and L-4 vertebra were mineralized at the temperature of 620 °C for 48 h and weighed. The mineralized bones were dissolved in 6 M HCl and then calcium and phosphorus content in the bone mineral content were assayed by a colorimetric method (Pointe Scientific standard kit).

### Serum calcium and phosphorus measurement

Trace element concentrations were measured using an Agilent 7700x ICP-MS spectrometer equipped with a MicroMist nebulizer and a Scott double-pass spray chamber, using a multi-element method including all elements present in the calibration solution [19]. Prior to the analysis, plasma samples were diluted (1:50 v:v) with ultrapure water (1:20, v/v)and acidified with a mixture of 1% butanol, 0.04% Triton, and 2% nitric acid (1:1:1, v/v/v). Sample trace element concentrations were automatically calculated by the software employed (Mass Hunter, Agilent). Data were exported to Microsoft Excel and corrected for dilution. We used metal solutions with final concentrations of 0.10, 20, 30, 40 and 50 µg/L for external calibration of the system. The measured trace element levels were within the certified range (5%). Calcium and phosphorus were measured using ICP-MS.

### Data analysis

All data were analyzed by a one-way analysis of variance, and the differences between means were established by Duncan’s multiple-range test. The data represents means and standard deviations. The significant level of 5% (p < 0.05) was used as the minimum acceptable probability for the difference between the means. All groups were included in the statistical comparison.

## Results

During space missions, weightlessness is responsible for physiological changes, such as bone loss. The study of novel mediators of bone metabolism is essential to understand the physiological responses of bone tissue to microgravity.

### Effect of cordymin on body weight

**Fig. 1:**
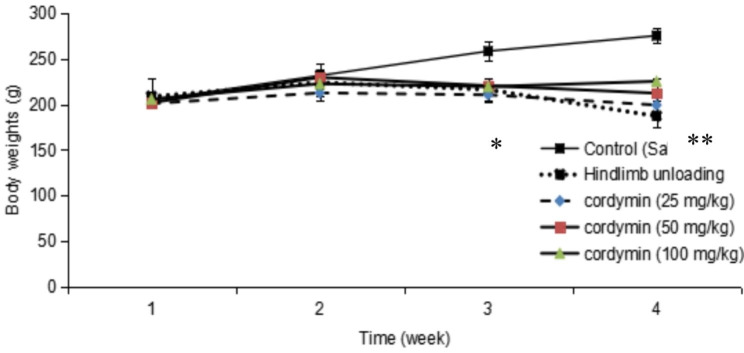
Body weight changes for the study. Hindlimb unloading significantly decreased body weights compared to untreated rats. Values are mean ± SEM. n = 10. *p < 0.05, ** p < 0.01compared to the control group at the same time point

Spaceflights-induced microgravity can alter various physiological processes in human’s body [20]. In the present study, we employed the hindlimb suspension rats model, a ground-based in vivo microgravity model that mimics physiological changes concomitant with space travel. The results of this study revealed that hindlimb unloading for 28 days significantly decreased body weight (Fig. 1). It is in agreement with other published studies [13]. The reduction in body mass was largely accounted for by reduced lean mass. Treatment with cordymin had significantly greater change in body weight compared with hindlimb unloading rats. However, the difference was not statistically significant.

### Effect of cordymin on mechanical testing

**Fig. 2:**
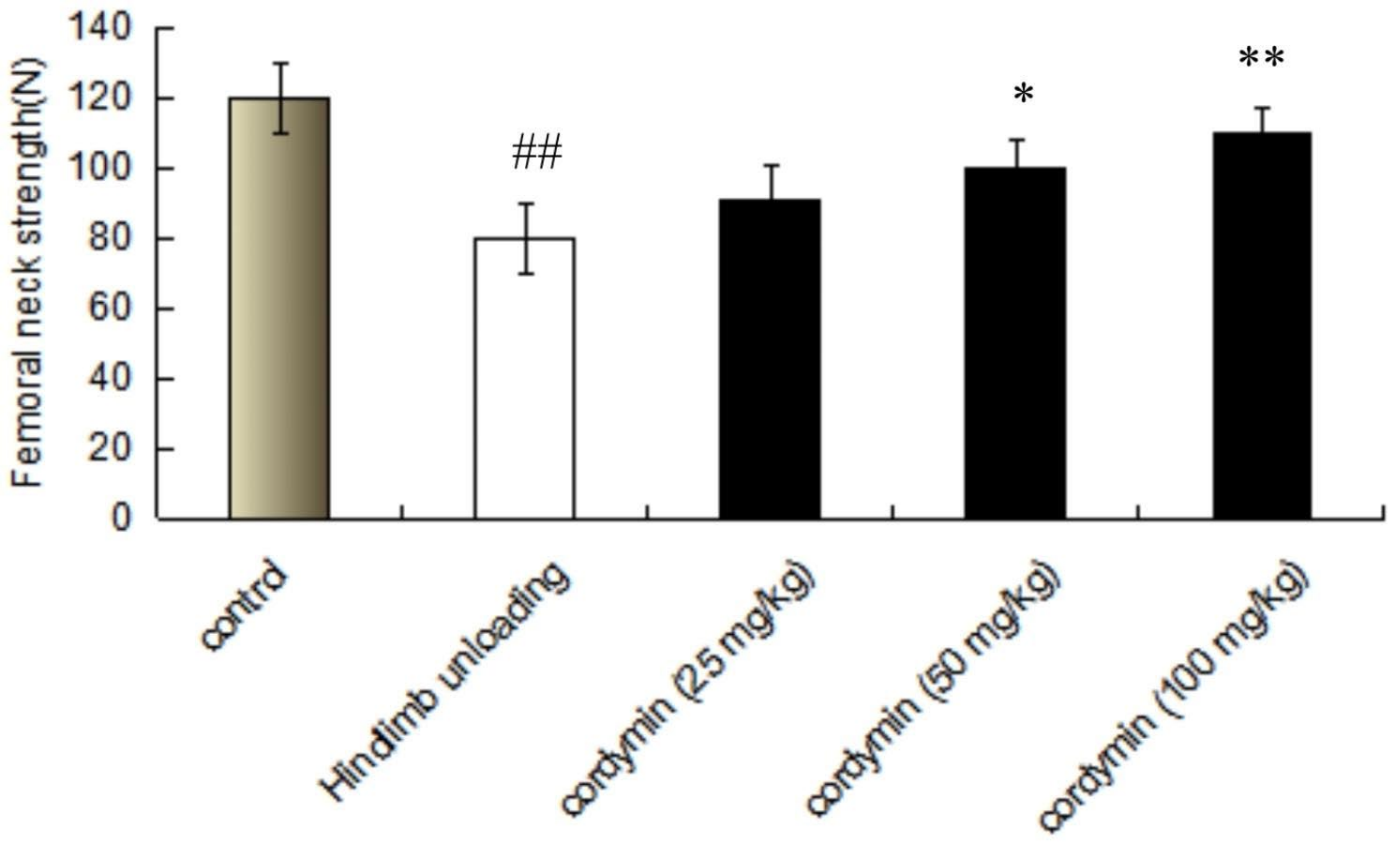
Mechanical strength of the femoral neck. The data are presented as mean ± SD (n = 10 per group). Values are mean ± SEM. n = 10. *p < 0.05, ** p < 0.01 compared to the hindlimb unloading.^##^ p < 0.01 compared to the control group

Evaluation of bone biomechanical properties is indispensable to determine the quality of bone, and the intensity of bone fracture directly correlates to the relationship between the structure of the bone and the strength and hardness of the bone. The average maximum fracture loading to the femoral necks was 30.1% lower in the hindlimb unloading rats compared with the control group (Fig. 2). Femoral neck strength in cordymin treated groups was higher than that in the hindlimb unloading group. These changes suggested long-term high-dose cordymin affected on bone mass or mechanical and properties.

### Effect of cordymin on Micro-CT

Wolff ’s Law states that a bone will adapt to the load under which it is placed [21]. Our results show that assessment of the femoral microarchitecture using microcomputed tomography revealed that cordymin had obvious effects on BV/TV, or Tb. Th in hindlimb unloading rats. After 4 weeks of cordymin treatment, trabecular bone volume of cordymin-100 group increased by 31.7% (P < 0.05) compared with the hindlimb unloading rats group, and this bone volume was similar to that of the control group (Table 1). Trabecular number of cordymin-100 treatment group was significantly lower than in the control group. However, trabecular thickness increased by 25.4% compared to the control group, increased by 47.8.7% compared with the hindlimb unloading group. Cortical thickness was 31.3% greater in cordymin-100 (P < 0.05) compared to hindlimb unloading group (Table 1). Cordymin-100 treatment partially ameliorated microgravity-induced deterioration of bone microarchitecture, as indicated by suppressing both the reduction in Tb.N and the increase in Tb.sp induced by hind limb unloading simulated microgravity.

**Table 1:** Bone structural variables of the fifth lumbar vertebral body by micro-CT (Mean ± standard deviation)

Groups	Trabecular bone volume (%)	Trabecular connectivity (1/mm^3^)	Trabecular number (1/mm)	Trabecular thickness (µm)	Structural model index	Cortical thickness (µm)
Control group	37.0 ± 5.1	48.3 ± 11.4	3.8 ± 0.3	101.5 ± 11.1	-0.38 ± 0.2	276 ± 40
Hindlimb unloading	27.1 ± 7.0^##^	39.1 ± 11.2^#^	3.4 ± 0.2^#^	87.0 ± 11.6^##^	0.08 ± 0.4^##^	249 ± 29^#^
Cordymin-25	29.0 ± 8.0	38.6 ± 11.0	3.7 ± 0.7	85.0 ± 10.4	0.09 ± 0.5	248 ± 28
Cordymin-50	31.9 ± 7.0^*^	38.8 ± 12.0	3.2 ± 0.5	101.1 ± 10.5^*^	0.08 ± 0.4	283 ± 28
Cordymin-100	39.0 ± 3.8^**^	37.6 ± 7.0	3.9 ± 0.5	121.2 ± 12.0^**^	-0.54 ± 0.4	300 ± 18

The data are presented as mean ± SD (n = 10 per group). Values are mean ± SEM. n = 10. *p < 0.05, ** p < 0.01 compared to the hindlimb unloading.^#^ p < 0.05 compared to the control group, ^##^ p < 0.01 compared to the control group

### Effect of cordymin on histological sections of the bone marrow

**Fig. 3:**
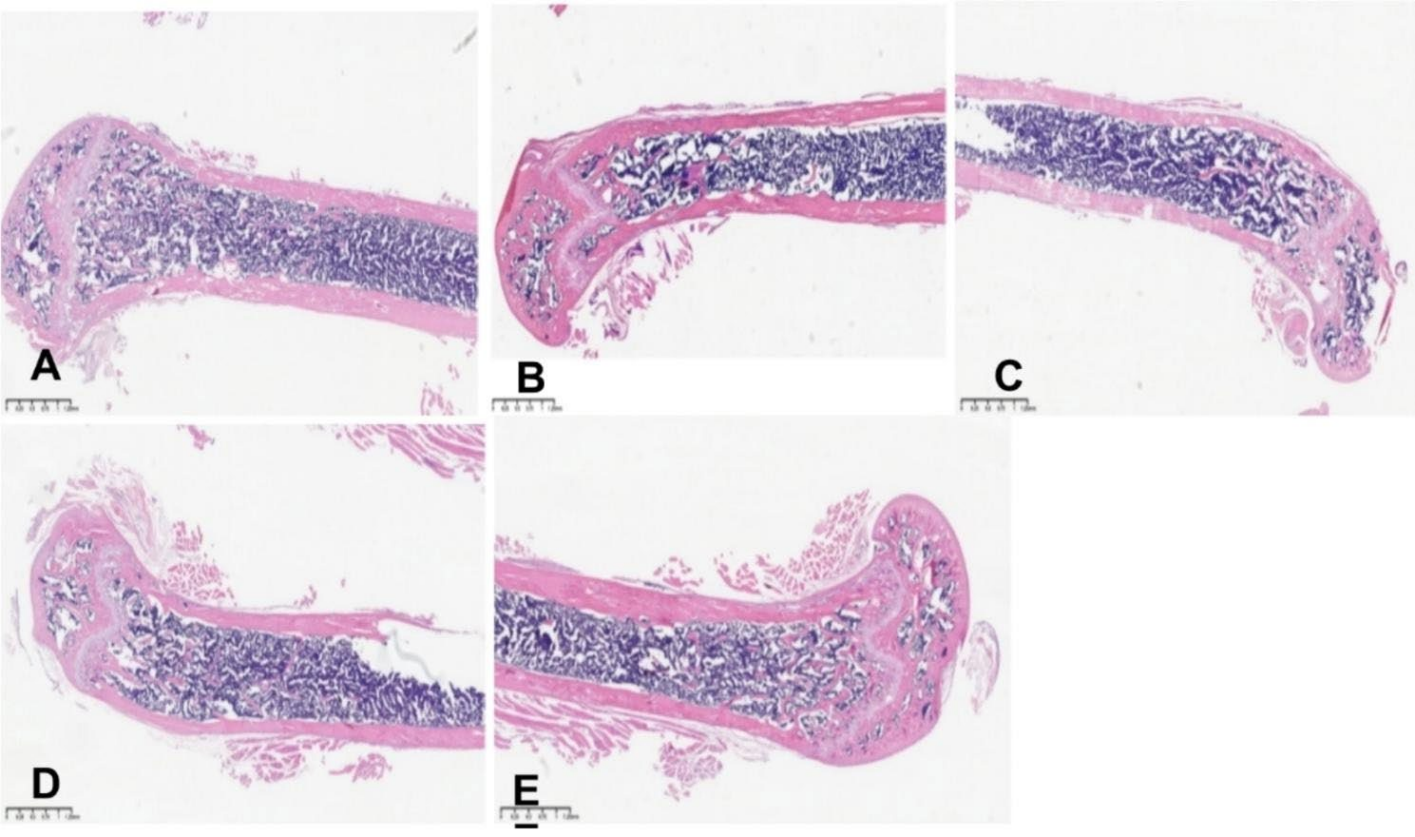
Effect of cordymin on the bone trabecula and intertrabecular spaces in rats under long term hindlimb unloading treatment. (**A**) Control group, (**B**) Hindlimb unloading group. (**C**) Cordymin-25group, (**D**) Cordymin-50 group, (**E**) Cordymin-100 group

Figure 3 shows histological sections of the bone marrow (BM) trabecula and intertrabecular spaces (A–E). Sections from femurs from the control group showed normal bone tissue with parallel arrangement of bone trabeculae (Fig. 3A). In contrast, the trabecular bone in rat femurs of the hindlimb unloading group became thinner and irregular, and the bone trabecular reticulate structure was destroyed (Fig. 3B). Meanwhile, rats received cordymin showed thick bone trabeculae (Fig. 3C, D, E).

### Effect of cordymin on gut microbiome

The emerging evidence continues to support the importance of the gut-bone axis in maintaining skeletal health [22]. However, there is few studies show the correlation between gut microbiome composition and bone loss shaped by microgravity. Animal limb unloading model is commonly used to imitate osteoporosis induced by microgravity [23]. To address the impact of hindlimb unloading on the gut microbiome, changes in the gut microbiome were evaluated using 16 S rRNA gene sequencing.

**Fig. 4:**
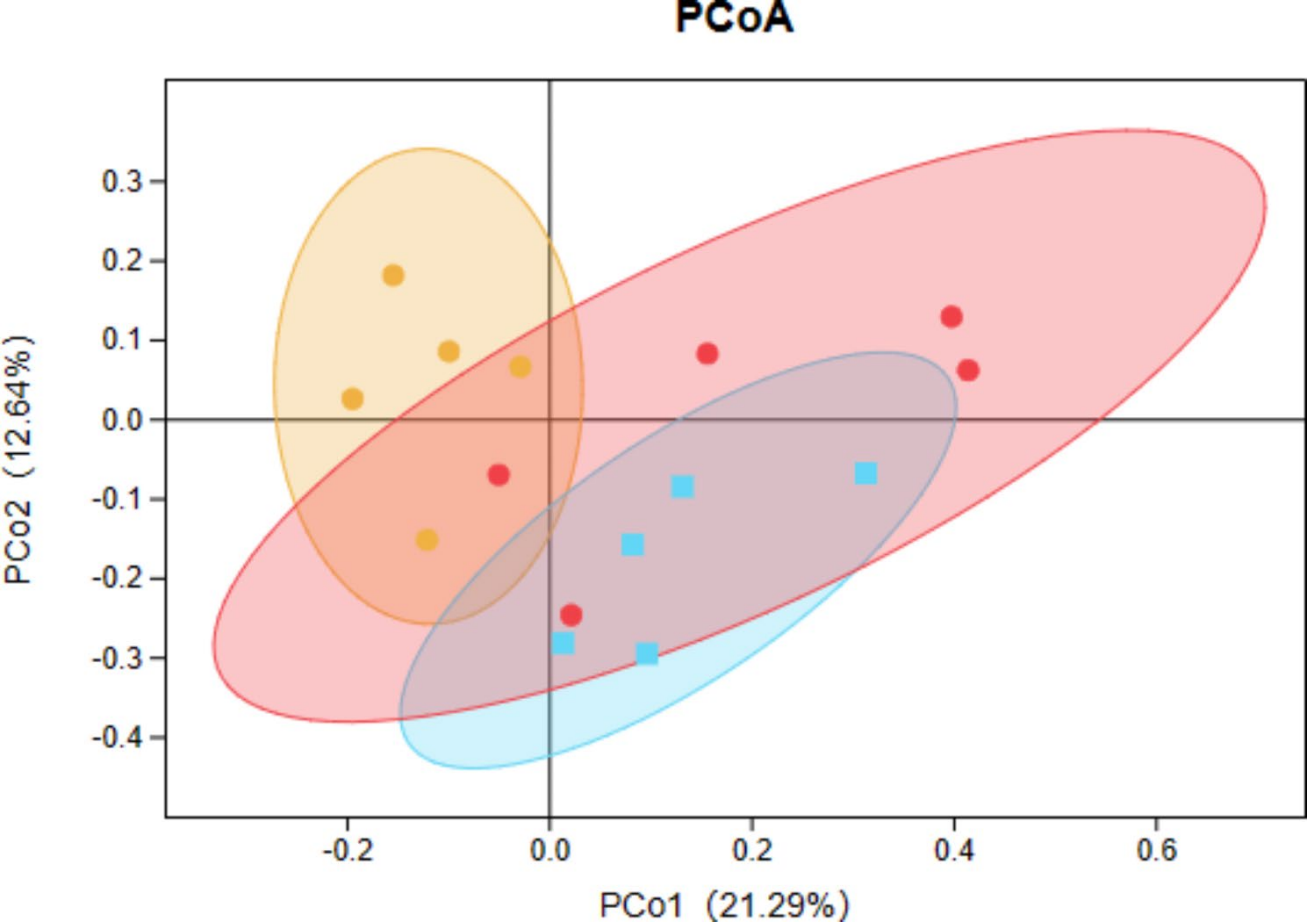
Principal component analysis (PCA) score plot of cordymin interventions on gut microbiota (Red is control group;Yellow is hindlimb unloading group, blue is cordymin-50 groups)

The gut bacterial communities of untreated and hindlimb unloading rats were different as shown in PCA analysis plot (Fig. 4). According to the PCA analysis plot in beta diversity analysis showed that the bacterial communities of control group and hindlimb unloading group were separated from each other significantly. However, cordymin-50 group were distinguished from the hindlimb unloading group. Furthermore,cordymin-50 group and control group were closer than hindlimb unloading group, suggesting that the microbial community structure of cordymin-50 group was related more closely to that of the control group than that of the hindlimb unloading group. These data demonstrated that interventions in cordymin could regulate the gut microbiota structure disrupted by hindlimb unloading.

**Fig. 5:**
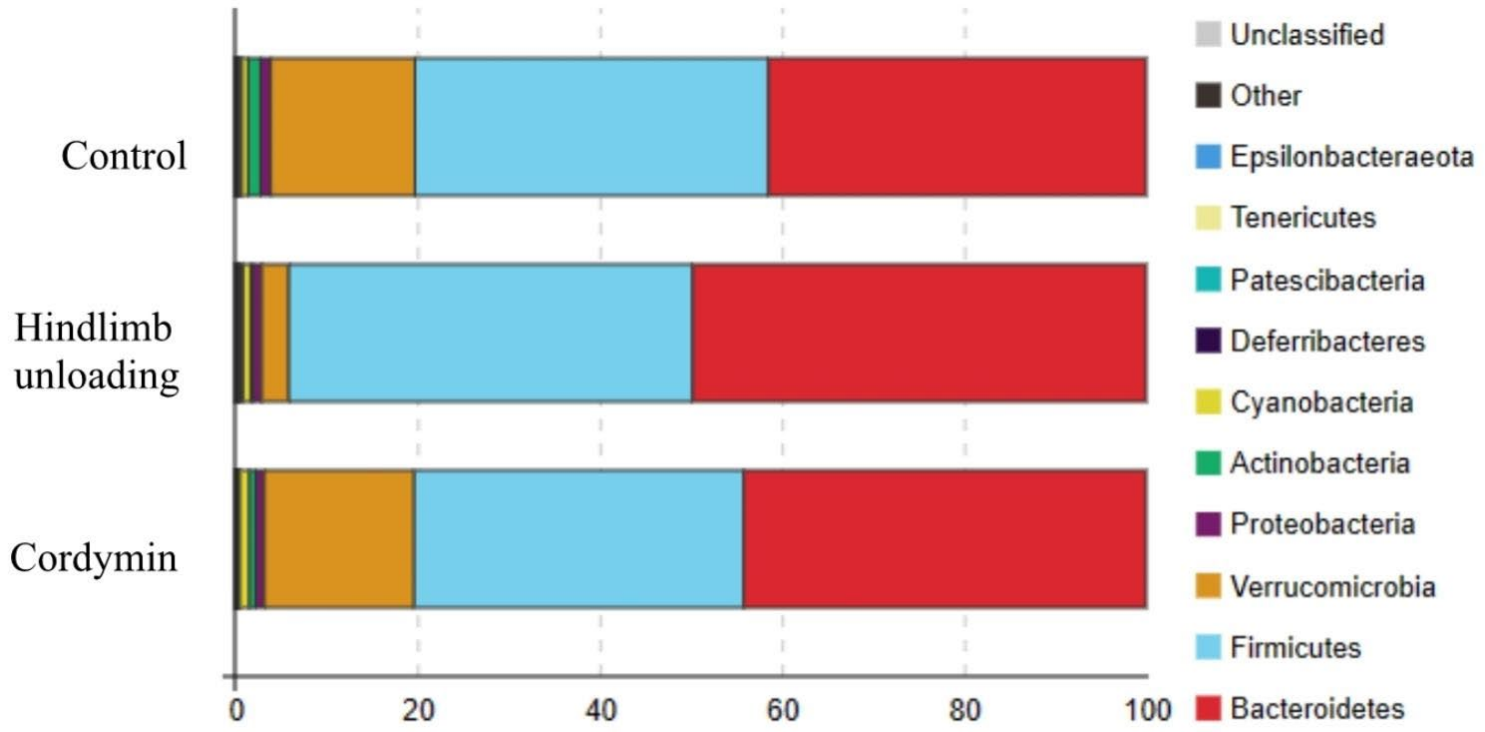
Analysis of differential composition in each group at Phylum level

The relative abundance of bacterial species in the 3 groups at the phylum level are shown in Fig. 5. At the phylum level, compared with the control group, the relative abundance of Firmicutes and Bacteroidetesin in the hindlimb unloading group increased, whereas the relative abundance of Verrucomicrobia and Acidobacteriota in the hindlimb unloading group decreased, indicating that hindlimb unloading led to the disturbance of gut microbiota in rats. However, hindlimb unloading rats receiving cordymin were characterized by decreased proportions of Firmicutes and Bacteroidetesin as well as increased proportions of Verrucomicrobia and Acidobacteriota compared to levels in hindlimb unloading rats.

### Effect of cordymin on trace elements

Pull of gravity is primarily mechanical forces normally acting on bone. If this forces is reduced, bone mineral content is affected. The relationship between bone mass and bone turnover was revealed in the Ca, P. In this study we showed that calcium lost from bone resorption decreases bone strength. This effect of microgravity on calcium homeostasis occurs rapidly and persists throughout a spaceflight [24]. The calcium content in the hindlimb unloading in the rats was 326.65 ± 21.443 mg/g. It is significantly reduced compared to the results obtained from the control group (403.44 ± 29.20 mg/g) (Table 2). Dramatic reduction in intestinal calcium absorption promotes bone resorption and the subsequent onset of osteoporosis [25]. However, the values of cordymin-50 and cordymin-100 treated group (369.40 ± 18.11 mg/dl and 389.32 ± 11.21 mg/dl respectively) were significantly higher than those of hindlimb unloading treated group(p < 0.05, p < 0.01).

**Table 2:** Bone mineral content of calcium (Ca) and inorganic phosphorus (P) (Mean ± standard deviation) (n = 10)

Different groups	Ca (mg/dl)	P (mg/dl)
Control group	403.44 ± 19.20	50.30 ± 14.13
Hindlimb unloading	326.65 ± 21.443^##^	45.30 ± 10.22
Cordymin-25	333.11 ± 12.70	46.11 ± 14.10
Cordymin-50	369.40 ± 18.11*	49.22 ± 12.21
Cordymin-100	389.32 ± 11.21**	50.31 ± 13.14*

*p < 0.05, ** p < 0.01 as compared with the hindlimb unloading group.^##^ p < 0.01 compared to the control group

There is accumulated evidence that phosphate binds to collagen fibrils in vitro in a number of stable complexes, the formation of collagen-orthophosphate bonds may be an important mechanism in the nucleation of calcium and phosphate as apatitc crystals in ostcoid [26]. The phosphate content in the hindlimb unloading in the rats was 45.30 ± 14.56 mg/g. It is significantly reduced compared to the results obtained from the control group (50.30 ± 14.13 mg/g) (Table 2). Furthermore,the values of cordymin-100 treated group (50.31 ± 13.14/dl respectively) were significantly higher than those of hindlimb unloading treated group.

At the same time, treatment with hindlimb unloading significantly increased the plasma total calcium and phosphorus concentrations. The effects of treatment with hindlimb unloading on plasma total calcium and inorganic phosphate concentrations are shown in Table 3. Hindlimb unloading rats had mean values for serum calcium and serum phosphorus (12.88 ± 0.5 mg/dl and 9.11 ± 0.6 mg/dl respectively). These values were significantly highly than those of control rats(9.11 ± 0.5 mg/dl and 4.88 ± 0.3 mg/dl respectively). It is reported that after the first 24 h of spaceflight, serum phosphate and calcium all show increased levels [27]. It is consistent with the findings of our results. Hindlimb unloading show a similar, possibly less, bone loss compared to spaceflight, calcium absorption were decreased, and serum ionized Ca was increased. Similarly,the cordymin-100 treated rats showed significantly decreased the mean values for serum calcium and serum phosphorus (9.21 ± 0.4 mg/dl and 5.36 ± 0.3 mg/dl respectively).

**Table 3:** Plasma concentrations in samples of calcium (Ca) and inorganic phosphorus (P) (Mean ± standard deviation) (n = 10)

Different groups	Ca (mg/dl)	P (mg/dl)
Control group	9.11 ± 0.5	4.88 ± 0.3
Hindlimb unloading	12.88 ± 0.5^#^	8.91 ± 0.6^##^
Cordymin-25	12.22 ± 0.1	8.77 ± 0.2
Cordymin-50	10.10 ± 0.6	6.23 ± 0.1
Cordymin-100	9.21 ± 0.4**	5.36 ± 0.3 *

*p < 0.05, ** p < 0.01 as compared with the hindlimb unloading group.^#^ p < 0.05 compared to the control group, ^##^ p < 0.01 compared to the control group

## Discussion

Hindlimb unloading model has been adapted to simulate the effects of microgravity on the musculoskeletal system, as experienced by astronauts during space fight [28]. However, whether “Gut-Microelements-Bone axis” is involved in mechanical stretching-mediated bone formation has been unclear. In the present study,we provides the first evidence that cordymin, a peptide purified from the Cordyceps sinensis, inhibits bone loss and osteoporosis in rats after hindlimb unloading. As expected, these findings was associated with significant improvements of gut microbiome and microelements.

In our study, hindlimb unloading treatment generated a significant decrease of mechanical strength in the femoral neck. Maximum load reflect the structural biomechanical parameters which would be influenced by the shape and size of the bone. The actual effect of the treatment on biomechanical competence could be fully evaluated with resort to the geometric properties reflecting the material biomechanical parameters. Decreased values of biomechanical parameters also indicated the reduced strength and stiffness of the femur due to hindlimb unloading. Treatment with cordymin led to a beneficial effect in the setting of unloading, as evidenced by increases in femoral strength values in the hindlimb unloading group. Micro-CT assay also demonstrated the deterioration of trabecular bone in the femur after hindlimb unloading treatment, which may be directly reflected from the histomorphometry analysis of cancellous bone volume. These results agreed with the findings reported in previous studies [29] that a fast decrease in BMD of cancellous bone occurs following hindlimb unloading. As expected,the treatment of cordymin for 28 continuous days during tail suspension could effectively attenuate the deterioration in femoral and tibiae trabecular bones. Cordymin treatment could restore the femur and tibia mechanical strength compared with hindlimb unloading rats. The microarchitectural properties were also improved after the cordymin administration including the increased BV/TV, Tb. Th, Tb.N. Therefore, it is highly possible that cordymin is capable of preventing weightlessness-induced bone loss.

The gut microbiota-bone axis is a relationship between the various metabolites that are produced by the microbes and the skeletal homeostasis [30]. The absorption of bone formation related nutrients can be regulated by gut microbiota. Dietary intake of peptide is fermented by gut microbiota, which produced SCFAs will benefit bone metabolism through increasing various vitamins and minerals, including vitamin K and B12, calcium, and magnesium [31]. The effects of the cordymin on the gut microbiota composition in hindlimb unloading rats were investigated by the 16 S rRNA sequencing. Our results showed that cordymin treatment extremely decreased proportions of Firmicutes and Bacteroidetesin as well as increased proportions of Verrucomicrobia and Acidobacteriota compared to levels in hindlimb unloading rats. The finding is partly consistent with the literature [32]. As for pathologic state, studies have shown that gut microbial dysbiosis is one of the risk factors of skeletal. The alternations of gut microbiota composition were varied among these studies maybe due to different diet, model, geographical location, and genetic background. However, the current work supported that the gut microbiota may be a novel therapeutic target in treatment of weightlessness-induced bone loss.

Recent studies have produced much new information on microbiota–mineral interactions. The gut microbiota is a major regulator of bone mineral density (BMD)in mice by altering the immune status in bone and affecting osteoclast-mediated bone resorption [33]. Gut microbiota can increase Ca^2+^ absorption and modulate the production of gut serotonin to regulate bone metabolism [34]. Interestingly,this study demonstrates that administration of cordymin to the hindlimb unloading animals increased significantly the calcium content in the examined bones as well as decreased plasma total calcium concentrations in hindlimb unloading rats. One possible reason is that cordymin treatment extremely decreased proportions of Firmicutes and Bacteroidetesin [35].

Several studies suggested that changes in dietary phosphorus could influence intestinal microbial composition. On the other side, gut microbiota stores phosphate and decrease the phosphate ion burden of the gut epithelium [36]. Based on current evidence,this study demonstrates that plasma inorganic phosphate concentrations in hindlimb unloading rats decreased after treatment with cordymin. Altought the precise molecular mechanisms for the phosphate - microbiota crosstalk remain unclear, Bone mineralization enhanced by changes in gut microbiota has also been proven in many reports [34, 36].

The administration of cordymin to the hindlimb unloading animals increased significantly the mineral content in the examined bones when compared to the hindlimb unloading group. These results remind us that mineral homeostasis is a balance between intestinal absorption and an internal contribution from the bone. Increased circulating concentrations of short-chain fatty acid(SCFA)may interact with the skeleton to activate bone forming osteoblasts, thus increasing bone mass and preventing bone loss. The positive effect of gut microbiota on mineral bioaccessibility and bioavailability may serve as an essential target for bone health and osteoporosis research.

## Conclusion

This study systematically evaluated the protective effects of cordymin on osteoporosis induced by hindlimb unloading in rats. The current study firstly demonstrated the in vivo osteoprotective effect of RSE. It could effectively reverse the deteriorated condition of bone trabecular in femur and tibiae induced by hindlimb unloading treatment. The results suggested the therapeutic effect of cordymin as an alternative supplement to be applied in the prevention and treatment of weightlessness-induced osteoporosis. Moreover, our data demonstrated that the bone protective effects of cordymin might be due to its direct influence the “gut - microelements - bone axis”. Meanwhile, a novel thought for the establishment of prevention and treatment goals.

The limitation of the present study was that there was no direct evidence for the modulating effects of cordymin on bone metabolism. The precise molecular mechanisms for the gut - microelements - bone crosstalk remain unclear. However, these data indicate that the cordymin might exert bone protective effects indirectly via modulating the complex relationship between gut microbiota, microelements and bone loss.

## Data Availability

The datasets used and/or analysed during the current study are available from the corresponding author on reasonable request.
