# Correction: Cordymin alleviates osteoporosis induced by hindlimb unloading via regulating the gut - microelements -bone axis --for non-clinical studies

**DOI:** 10.1186/s12891-023-07154-7

**Published:** 2024-01-23

**Authors:** Wei Qi, Tiancheng Ma, Yufei Ji, Hong Jia, Qiang Sun, Dawei Zhang

**Affiliations:** 1 Department of Orthopaedics, Xijing Hospital, The Air Force Medical University, Xi’an, 710032 China; 2 Xi’an, China

**Correction: BMC Musculoskelet Disord 24, 932 (2023)**

10.1186/s12891-023-07057-7

Following publication of the original article [1], the authors identified an error in the author name of Dawei Zhang. The name should read Dawei Zhang and not Danwei Zhang. Qiang Sun has been corrected as the co-corresponding author instead of Wei Qi. And Qi Wei was the first author.

The original article [1] has been corrected.
